# DISPARITIES AND TRENDS IN COMPREHENSIVE PAIN MANAGEMENT AMONG THE ADULT POPULATION IN THE POST-OPIOID EPIDEMIC

**DOI:** 10.1093/geroni/igad104.3121

**Published:** 2023-12-21

**Authors:** Sebastián Spataro, Molly Candon

**Affiliations:** University of Pennsylvania, Philadelphia, Pennsylvania, United States; University of Pennsylvania, Philadelphia, Pennsylvania, United States

## Abstract

**Introduction:**

Chronic pain is an epidemic, but there are various therapies that are effective at treating it. Disparities in access to chronic back pain management are well documented. However, studies have largely focused on the gap in utilization between Black and White patients, and on the use opioid therapies. The extent of disparities in utilization for other groups and how disparities by demographics interact with socioeconomic characteristics, are unclear.

**Methods:**

Using insurance claims from Optum Data, we identified patients who had chronic low back pain between 2010- 2020. We measured trends in pharmacological and nonpharmacological therapies, disaggregating measures by sex, age, race/ethnicity, educational attainment, income, and parenthood. We estimated linear probability models to understand which demographic and socioeconomic characteristics were most predictive of utilization.

**Results:**

There were large differences in the utilization of pharmacological and nonpharmacological pain care, with surprising findings. First, we found that Black patients were the most likely to be prescribed opioids, as well gabapentin, muscle relaxers, and NSAIDs, followed by White patients, Hispanics; Asians were the least likely to engage in pharmacological therapy. Linear probability models indicated that the largest predictor of pharmacological and nonpharmacological pain management was education and income.

**Discussion:**

We document disparities in pain management, which point to important policy implications. Preferences of pain management warrant further attention, as our findings suggest that income and education are the largest driver. It could be that the time and out-of-pocket costs associated with nonpharmacological pain management reduce the likelihood that patients opt for these therapies.

